# Partial thickness subfoveal hole in a patient treated with tamoxifen: a case report and review of the literature

**DOI:** 10.1186/s13256-022-03681-4

**Published:** 2022-12-21

**Authors:** Ashley Sohn, George Sanchez, Dimosthenis Mantopoulos

**Affiliations:** 1 Geisel School of Medicine at Dartmouth, Hanover, NH USA; 2 Department of Ophthalmology, Dartmouth-Hitchcock Medical Center, One Medical Center Drive, Lebanon, NH 03756 USA

**Keywords:** Tamoxifen, Macular hole, Macular cyst, Retinal toxicity

## Abstract

**Background:**

We describe a patient presenting with a partial thickness subfoveal hole in the right eye after tamoxifen treatment for breast cancer.

**Case presentation:**

A 76-year-old Caucasian female presented with a 1-day history of acute central scotoma and blurry vision in the right eye. The patient had been receiving oral tamoxifen for 5 years as adjuvant treatment for stage I lobular breast cancer. Her past ocular history was significant for complete, uneventful, and bilateral posterior vitreous detachment. Clinical examination and optical coherence tomography revealed a new, partial thickness subfoveal hole sparing the inner retinal layers. Observation was recommended. At the last follow-up examination, 1 year after the initial presentation, the subfoveal hole remained stable and visual acuity remained stable.

**Conclusion:**

Tamoxifen has been associated with a plethora of ophthalmic adverse events, including macular holes, some of which are partial thickness subfoveal holes. Holes with this almost unique morphology are uncommon, and eye care professionals should be aware of this association given the frequency of tamoxifen use, as well as the low success rate of surgical repair with pars plana vitrectomy.

## Background

Tamoxifen is a selective estrogen receptor modulator (SERM) used as adjuvant endocrine therapy for hormone receptor-positive breast cancer [1]. Previously reported ocular side effects include retinal crystals, optic neuritis, macular edema, dry eye, and cataracts [1, 2]. The retinal crystals used to be one of the classic ocular complications of tamoxifen in patients treated with 100 mg daily. Fortunately, these crystals have now become less common thanks to the protocols that recommend treatment with up to 20 mg/day for no more than 5 years [2]. Aside from these side effects, outer macular holes secondary to tamoxifen use have also been reported, and the pertinent literature is summarized in Table 1. Macular holes are an uncommon adverse event that can occur with tamoxifen, a commonly used drug.

**Table 1:** Summary of tamoxifen-related macular hole case reports in the current literature

Author	Year of publication	Relevant history (age and cancer history)	Total tamoxifen given	Presenting symptoms	OCT findings
Gualino* et al*.	2005	Case 1: 64-year-old woman with history of breast cancer status-post (s/p) mastectomy	34.2 g	N/A	Foveolar cystoid space with focal disruption of the photoreceptor line OU
		Case 2: 72-year-old woman with history of breast cancer	30.6 g	N/A	Foveolar cystoid space with focal disruption of photoreceptor line in OD, with no macular thickening
Chung* et al*.	2010	51-year-old woman s/p radical mastectomy for invasive ductal breast cancer 7 years prior	0.9 g	Decreased visual acuity	Focal defect of outer retinal layer in the fovea OS Normal OD
Caramoy* et al*.	2011	49-year-old woman with history of breast cancer s/p mastectomy 6 years prior	33 g	11 months of visual acuity deterioration, central scotoma in OS	Defect in the outer retinal layer with sharp edges; posterior hyaloid still attached
Georgalas* et al*.	2013	55-year-old woman with history of breast cancer, diagnosed 11 years prior	73 g	Gradual visual deterioration OU	Extensive areas of disruption in inner retinal layers OU
Doshi* et al*.	2014	Case 1: 63-year-old woman with hx of stage 1 breast cancer s/p mastectomy 14 years prior	36.5 g	Difficulty with reading	Cavitary spaces with disruption of the outer retinal bands OS > OD
		Case 2: 50-year-old woman with history of breast cancer s/p lumpectomy and radiation 3 years prior	18.25 g	Distortion in OS for 1 year	Thinning of inner retina with draping of ILM OD and near full-thickness cavitation OS
		Case 3: 52-year-old woman with history of breast cancer s/p bilateral mastectomy with chemotherapy and radiation 8 years prior	58.4 g	Blurred vision and central scotoma OS for 1 year	Cavitary space OS
Hu* et al*.	2016	53-year-old woman with history of ductal breast carcinoma s/p radical mastectomy and adjuvant chemotherapy 3 years prior	18 g	Gradual decrease of visual acuity OU for 3 months	Ruptured ellipsoid zone, granular change of outer segments of photoreceptors in OD Macular hole in OS
Torrell-Belzach* et al*.	2020	63-year-old woman with history of breast cancer diagnosed at age 41	73 g	Mild, progressive bilateral vision loss for years	Bilateral macular hole with a thin overlying roof of inner retinal remnant and small cystic spaces in the periphery of the hole Posterior hyaloid attached

*Hx* history,* ILM* inner limiting membrane,* OD* right eye,* OS* left eye,* OU* both eyes,* S/p* status post

## Case presentation

A 76-year-old Caucasian female presented with a 1-day history of acute central scotoma and blurry vision in the right eye. She denied trauma, strenuous physical activity, or Valsalva-like maneuvers around the symptom onset. Her ocular history was notable for remote, previously documented, bilateral posterior vitreous detachment (PVD) without vitreomacular traction. The past medical history was significant for stage I lobular breast cancer, diagnosed and surgically resected. Following lumpectomy and postoperative radiation, the patient was on adjuvant oral tamoxifen (20 mg daily, total dose 36.5 g) for 5 years. The patient was no longer taking tamoxifen at the time of presentation.

**Fig. 1:**
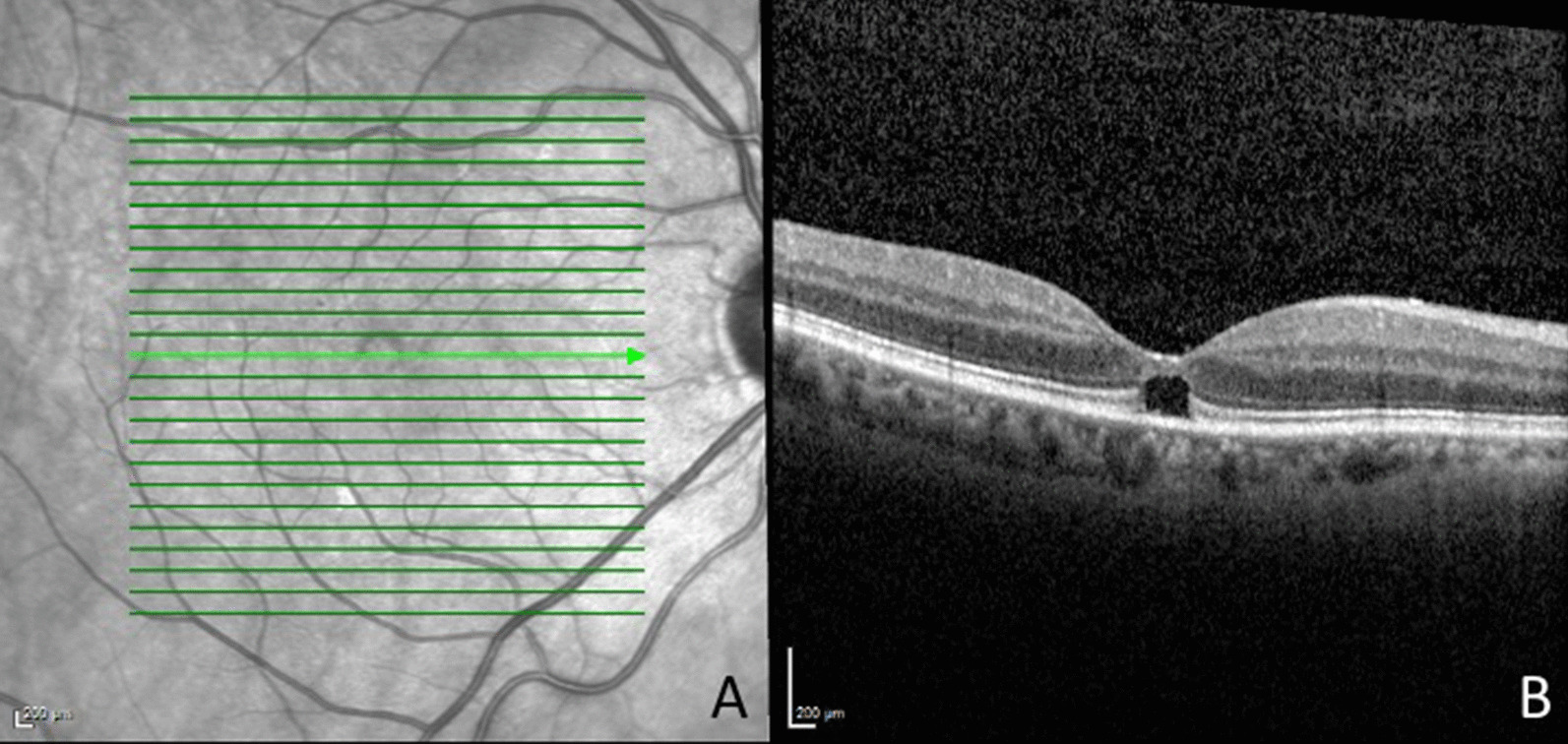
Near infrared image (**A**) of the right eye showing a hyporeflective round lesion in the fovea. Optical coherence tomography of the same eye (**B**) demonstrating a partial thickness, outer retinal hole with ellipsoid zone loss. Note that the inner retinal layers are preserved. The patient had a history of posterior vitreous detachment with no signs of vitreomacular traction in the vitreomacular interface

**Fig. 2:**
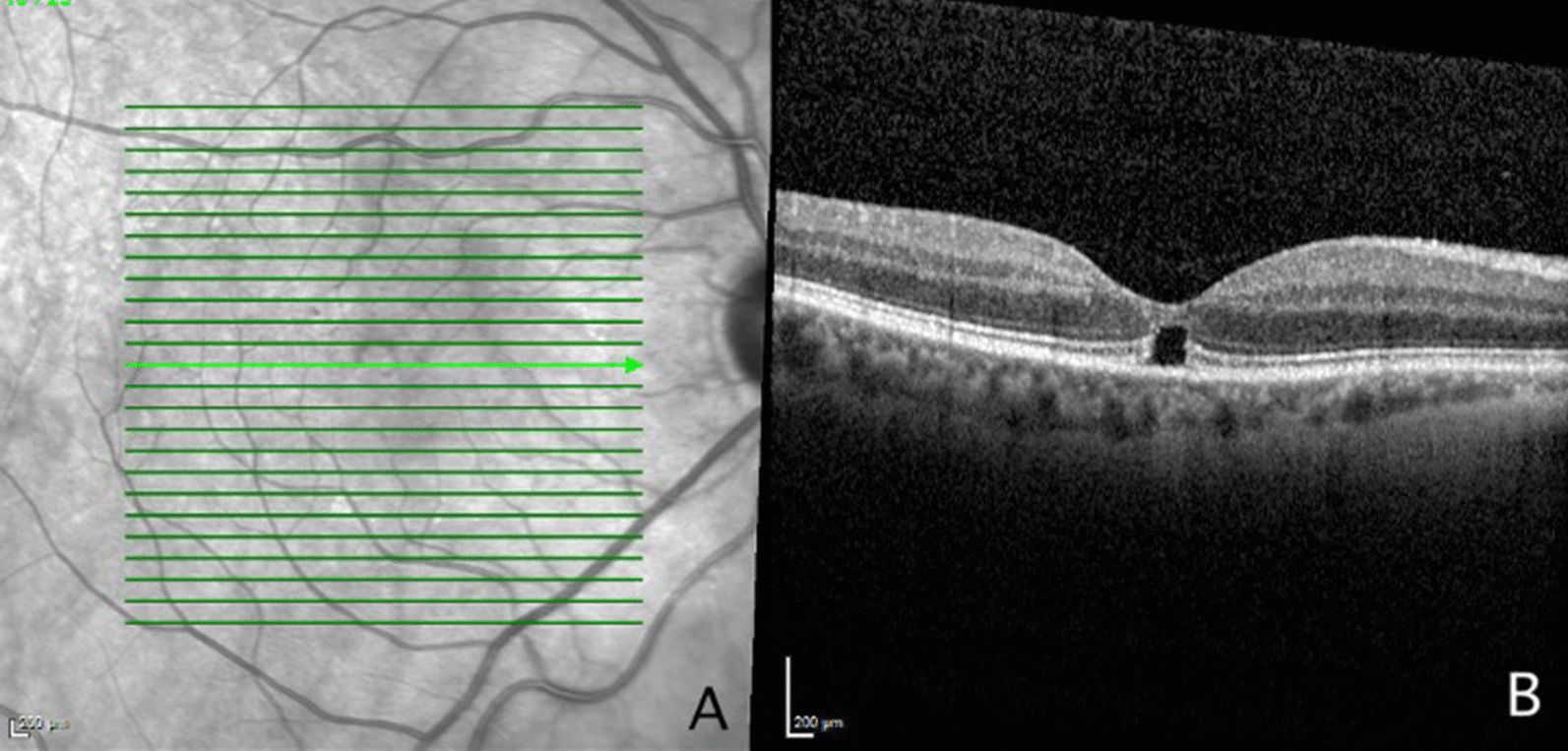
Near infrared image (**A**) of the right eye at latest follow-up visit. Optical coherence tomography (**B**) showed no anatomic changes since the initial presentation

**Fig. 3:**
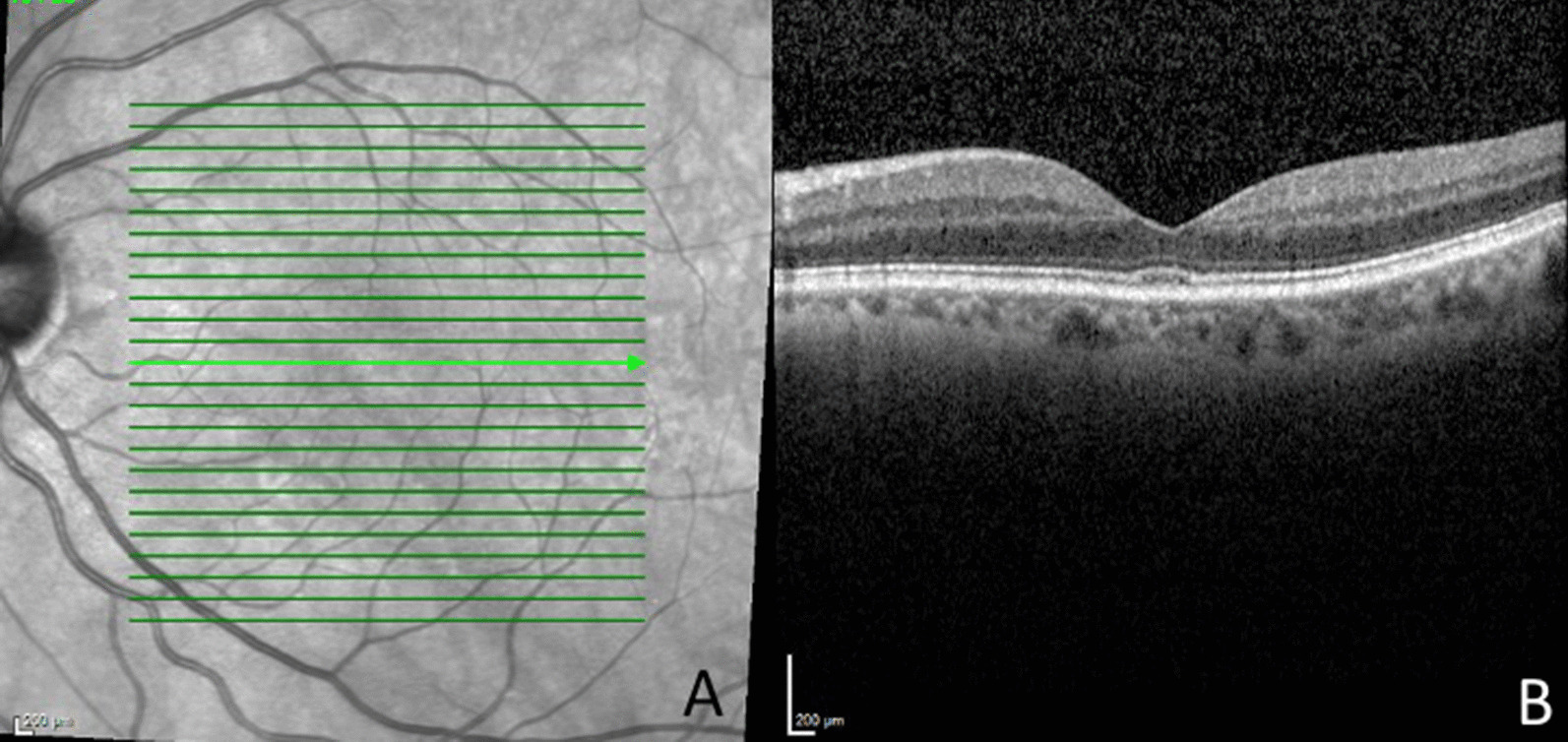
Near infrared image (**A**) of the left eye at latest follow-up visit. Optical coherence tomography (**B**) of the left eye was essentially normal

On examination, her best-corrected visual acuity was 20/40 OD and 20/25 OS. The anterior segment examination demonstrated bilateral 1+ nuclear sclerotic cataracts. On fundus examination, the right eye demonstrated a PVD, a pink optic nerve with cup/disc ratio of 0.3, normal vessels, and abnormal foveal reflex in the right eye. Infrared imaging and optical coherence tomography (OCT) of her right eye (Fig. 1) showed a partial thickness subfoveal hole with total disruption of the ellipsoid zone. The examination and OCT of the left eye was essentially normal. The patient was followed closely and the subfoveal cyst remained unchanged on OCT. At the latest follow-up visit, 1 year after the initial presentation, the best-corrected visual acuity in the right eye was 20/30 and there were no anatomic changes on OCT imaging (Fig. 2). OCT of the left eye at latest follow-up was essentially normal (Fig. 3).

## Discussion and conclusions

Previous case reports suggest an association between tamoxifen use and macular hole development. Cronin* et al*. [3] found that the risk for macular hole development was significantly higher in women treated with tamoxifen compared with the control group of the same age (4.12% versus 0.82%, *p* = 0.0001). Although most tamoxifen-related macular holes have the typical foveal configuration on OCT, some of them do not. Gualino* et al*. [4] first published two cases of tamoxifen-retinopathy associated with outer foveal cysts and photoreceptor disruption, while the inner retinal layers were spared. One year later, Martine* et al*. [5] reported a similar case and described the findings as “large, foveolar pseudo-cyst.” Since then, other authors have shared similar findings that, in some cases, were bilateral [6]. It has previously been hypothesized that this, almost unique, configuration is secondary to a neurodegenerative process of the Müller cells [6].

A variety of surgical approaches for management of tamoxifen-related macular holes has been previously described. Bernstein and DellaCroce published a case of bilateral sequential macular holes in a 65-year-old female treated with tamoxifen [7]. The right macular hole only closed after the second pars plana vitrectomy (PPV) with C_3_F_8_, while the left eye’s hole remained open after a single PPV with gas. Torrell-Belzach* et al*. also found that PPV with inner limiting membrane (ILM) peeling and SF_6_ gas tamponade was unsuccessful [8]. One possible explanation is that the pathophysiology of these holes does not include the typical centrifugal traction on the surface of the retina that usually resolves upon removal of the ILM of the macula followed by gas tamponade [8].

The ocular side effects of tamoxifen have been known to ophthalmologists and optometrists alike for decades [9–12]. Nevertheless, the guidelines regarding appropriate screening and management of ocular toxicity due to tamoxifen are not as clear as seen in other medications. In a case of clinically significant ocular toxicity, the management can be challenging. For that reason, the patient’s expectations should be carefully adjusted after a lengthy conversation about the risks, benefits, and alternatives of each option [1]. In this case, the macular hole did not progress after discontinuation of the causative agent; as such, early surgical intervention was not needed.

## Data Availability

All data generated or analyzed during this study are included in this article.

## Abbreviations

ILM: Inner limiting membrane
OCT: Optical coherence tomography
PPV: Pars plana vitrectomy
PVD: Posterior vitreous detachment
SERM: Selective estrogen receptor modulator
